# Slmb antagonises the aPKC/Par-6 complex to control oocyte and epithelial polarity

**DOI:** 10.1242/dev.109827

**Published:** 2014-08

**Authors:** Eurico Morais-de-Sá, Avik Mukherjee, Nick Lowe, Daniel St Johnston

**Affiliations:** The Gurdon Institute, The Department of Genetics, University of Cambridge, Tennis Court Road, Cambridge CB2 1QN, UK

**Keywords:** Axis formation, Par-1, SCF, Ubiquitylation, Apical-basal polarity, Oskar

## Abstract

The *Drosophila* anterior-posterior axis is specified when the posterior follicle cells signal to polarise the oocyte, leading to the anterior/lateral localisation of the Par-6/aPKC complex and the posterior recruitment of Par-1, which induces a microtubule reorganisation that localises *bicoid* and *oskar* mRNAs. Here we show that oocyte polarity requires Slmb, the substrate specificity subunit of the SCF E3 ubiquitin ligase that targets proteins for degradation. The Par-6/aPKC complex is ectopically localised to the posterior of *slmb* mutant oocytes, and Par-1 and *oskar* mRNA are mislocalised. Slmb appears to play a related role in epithelial follicle cells, as large *slmb* mutant clones disrupt epithelial organisation, whereas small clones show an expansion of the apical domain, with increased accumulation of apical polarity factors at the apical cortex. The levels of aPKC and Par-6 are significantly increased in *slmb* mutants, whereas Baz is slightly reduced. Thus, Slmb may induce the polarisation of the anterior-posterior axis of the oocyte by targeting the Par-6/aPKC complex for degradation at the oocyte posterior. Consistent with this, overexpression of the aPKC antagonist Lgl strongly rescues the polarity defects of *slmb* mutant germline clones. The role of Slmb in oocyte polarity raises an intriguing parallel with *C. elegans* axis formation, in which PAR-2 excludes the anterior PAR complex from the posterior cortex to induce polarity, but its function can be substituted by overexpressing Lgl.

## INTRODUCTION

In *C. elegans* and *Drosophila*, the anterior-posterior (AP) axis is defined through the formation of complementary cortical domains of PAR proteins in the zygote and oocyte, respectively (St Johnston and Ahringer, 2010). Polarity in *C. elegans* is induced by sperm entry and depends on an interaction between the sperm-derived centrosome and the posterior cortex of the fertilised egg (Cowan and Hyman, 2004; Cuenca et al., 2003; Goldstein and Hird, 1996; Tsai and Ahringer, 2007). This unknown signal from the centrosome or the centrosomal microtubules initiates polarisation by two mechanisms. First, it inactivates myosin contractility at the posterior of the zygote to trigger a contraction of the actomyosin cortex towards the anterior, and this localises the anterior PAR proteins PAR-3, PAR-6 and aPKC, allowing PAR-2 and PAR-1 to associate with the posterior cortex (Cheeks et al., 2004; Motegi and Sugimoto, 2006; Munro et al., 2004; Schonegg and Hyman, 2006). Second, the centrosomal microtubules deliver PAR-2 to the posterior cortex, where it recruits PAR-1 to induce removal of the anterior PAR proteins independently of cortical contraction (Motegi et al., 2011; Zonies et al., 2010). The complementary cortical domains are then maintained by mutual antagonism between the anterior and posterior PAR proteins: aPKC phosphorylates PAR-2 and PAR-1 to prevent their cortical localisation, and PAR-1 is thought to phosphorylate PAR-3 to exclude the anterior PAR complex from the cortex (Hao et al., 2006; Motegi et al., 2011). This system is buffered by Lgl (LGL-1 – WormBase), which localises to the posterior cortex like PAR-2, and, although non-essential, can rescue the *par-2* mutant phenotype when overexpressed (Beatty et al., 2010; Hoege et al., 2010).

Unlike *C. elegans*, the AP axis of *Drosophila* is defined before fertilisation through the polarisation of the developing oocyte. Each egg chamber in the *Drosophila* ovary is formed from a cyst of 16 germ cells, comprising the oocyte and 15 nurse cells, surrounded by a monolayer of somatic follicle cells (Bastock and St Johnston, 2008). The oocyte comes to lie posterior to the nurse cells and signals through Gurken to induce the adjacent follicle cells to adopt a posterior fate (Godt and Tepass, 1998; González-Reyes et al., 1995; González-Reyes and St Johnston, 1998; Roth et al., 1995; Torres et al., 2003). At stage 7 of oogenesis, these posterior follicle cells signal back to polarise the AP axis of the oocyte.

Although the nature of this polarising signal is unknown, it induces a very similar distribution of PAR proteins to that in the *C. elegans* zygote. Bazooka (Baz; *Drosophila* Par-3), Par-6 and aPKC disappear from the posterior cortex of the oocyte and mark its anterior and lateral sides, whereas Par-1 is recruited to the posterior cortex (Doerflinger et al., 2006, 2010). There is no orthologue of Par-2 in *Drosophila*, nor is there any evidence that the actomyosin cortex of the oocyte contracts anteriorly, making it unclear how polarity is established. However, once the asymmetric distribution of PAR proteins is established, it is maintained by mutual antagonism between the anterior Baz/Par-6/aPKC complex and posterior Par-1. aPKC phosphorylates Par-1 to exclude it from the cortex, and Par-1 phosphorylates Baz to disrupt its interaction with aPKC and its self-association (Benton and St Johnston, 2003; Doerflinger et al., 2010; Hurov et al., 2004; Suzuki et al., 2004). Lgl [L(2)gl – FlyBase] might also play a redundant role in AP polarity in *Drosophila*, as it does in *C. elegans*, since it localises to a crescent at the posterior of the oocyte like Par-1, and *lgl* mutant germline clones show reduced Par-1 localisation although they show no obvious polarity phenotypes (Li et al., 2008; Tian and Deng, 2008).

The cortical PAR proteins specify the AP axis by controlling the organisation of the oocyte microtubule cytoskeleton, so that microtubules are nucleated or anchored at the anterior and lateral cortex, but not at the posterior, where only plus ends are found (Cha et al., 2002; Clark et al., 1994, 1997; Theurkauf et al., 1992). This polarised microtubule network then directs the localisation of *bicoid* and *oskar* mRNAs to the anterior and posterior poles of the oocyte, respectively, where they define the AP axis of the embryo (Brendza et al., 2000; Weil et al., 2006; Zimyanin et al., 2008). How the PAR proteins organise the microtubules is unknown, but Par-1 seems to play a key role in this process by excluding microtubule minus ends from the posterior (Doerflinger et al., 2010). *par-1* mutants therefore develop a symmetric microtubule cytoskeleton, in which minus ends are found all around the oocyte cortex with the plus ends and *oskar* mRNA concentrated in the centre (Shulman et al., 2000; Tomancak et al., 2000).

In order to identify missing components in *Drosophila* AP axis formation, we have performed a large-scale germline clone screen for mutants that disrupt the posterior localisation of GFP-Staufen, an RNA-binding protein that associates with *oskar* mRNA (Martin et al., 2003). Here, we report our analysis of a lethal complementation group from the screen that disrupts the earliest step in the AP polarisation of the oocyte.

## RESULTS

### *slmb* mutants disrupt *oskar* mRNA localisation

**Fig. 1.**
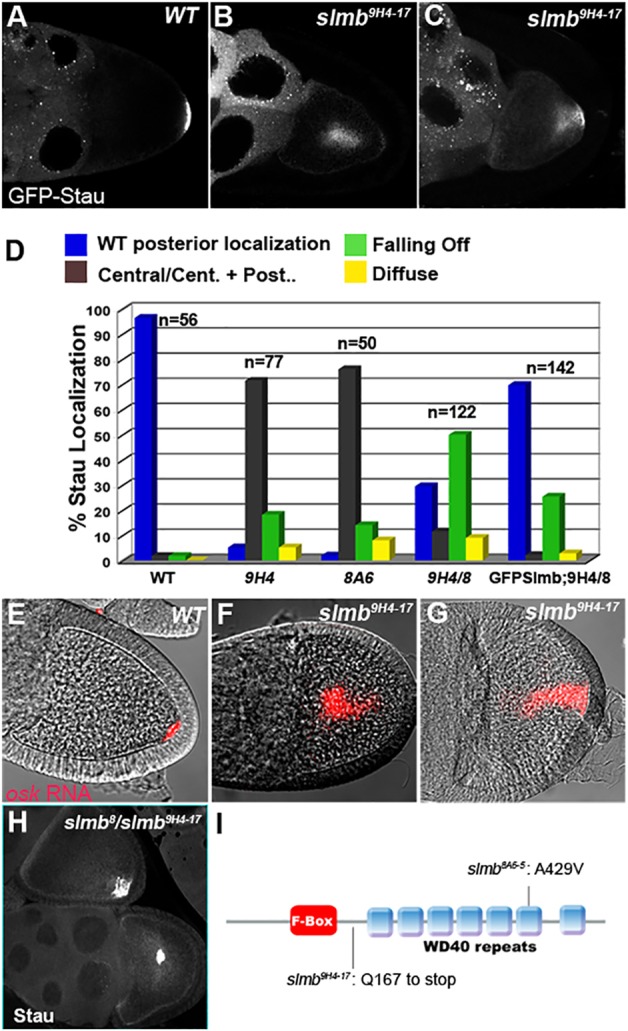
**Slmb is required for the localisation of *oskar* mRNA.** (A-C) GFP-Stau localisation in wild-type (A) and *slmb*^9H4-17^ *Drosophila* germline clones (B,C). GFP-Stau localises to the middle of most *slmb* mutant oocytes (B), but sometimes appears to be falling off from the posterior cortex (C). (D) The quantification of Stau localisation in stage 9-11 oocytes in: (1) *w^–^* (WT); (2) *slmb*^9H4-17^ germline clone (9H^4^); (3) *slmb*^8A6-5^ germline clone (8A^6^); (4) hs-Slmb; *slmb*^8^/*slmb*^9H4-17^ (9H4/8); and (5) *nanos-Gal4VP16*; UASGFP-Slmb; hs-Slmb, *slmb*^8^/*slmb*^9H4-17^ (GFPSlmb;9H4/8). (E-G) *In situ* hybridisations for *oskar* (*osk*) mRNA. *osk* mRNA (red) localises to the posterior of wild-type oocytes (E), but is found in the middle (F) or extending from the posterior (G) of *slmb*^9H4-17^ germline clone oocytes. (H) Example of the variable Stau localisation phenotype of hs-Slmb, *slmb*^8^*/slmb*^9H4-17^. (I) Molecular characterisation of *slmb* alleles. *slmb*^9H4-17^ contains a nonsense mutation at amino acid 167. *slmb*^8A6-5^ has an alanine-to-valine substitution at amino acid 429. The F-box domain and the WD40 repeats of Slmb are shown.

**Fig. 2.**
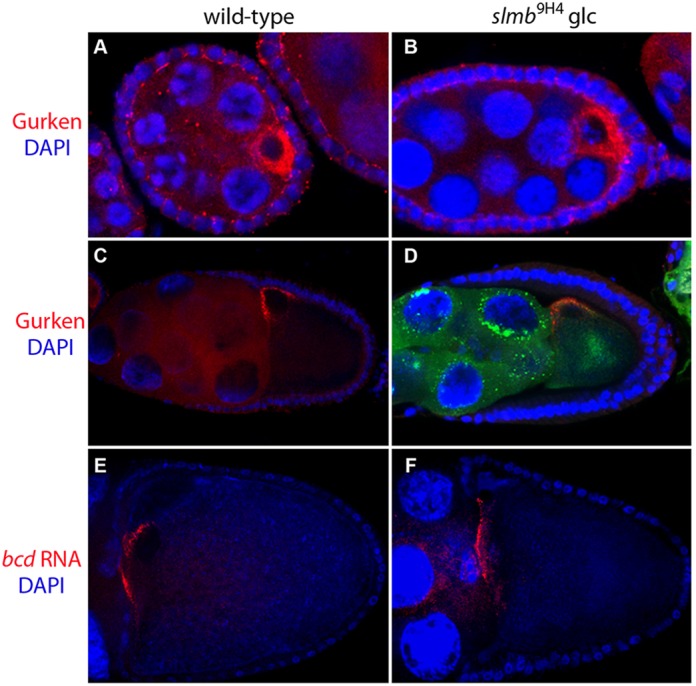
***slmb* mutants do not affect Gurken and *bicoid* mRNA localisation.** (A,B) Localisation of Gurken protein in stage 6 egg chambers. Gurken is expressed at the posterior of the oocyte at this stage to signal to the adjacent follicle cells. Gurken localisation is normal in *slmb*^9H4-17^ mutant clones. (C,D) Gurken accumulates at the dorsal-anterior corner of stage 9 oocytes in wild-type (C) and *slmb*^9H4-17^ (D) egg chambers. Note the ectopic GFP-Staufen localisation (green in D) in the middle of the mutant oocyte. (E,F) *In situ* hybridisations for *bicoid* (*bcd*) mRNA (red). *bcd* mRNA is localised to the anterior cortex of the oocyte in wild type (E) and in *slmb*^9H4-17^ mutants (F). DNA is marked with DAPI.

The *wellman* locus is a lethal complementation group of two alleles, *wel*^8A6-5^ and *wel*^9H4-17^, that was identified in the GFP-Staufen germline clone screen on chromosome 3R (Martin et al., 2003). In more than half of *wel*^8A6-5^ and *wel*^9H4-17^ homozygous mutant germline clones, GFP-Stau is mislocalised to the centre of the oocyte at stage 9, rather than to the posterior pole as in wild type (Fig. 1A,B,D). The remaining mutant oocytes display a milder phenotype, in which GFP-Staufen localises at both the posterior and centre of the oocyte or extends from the posterior cortex into the cytoplasm (Fig. 1C). To confirm that this phenotype reflects a defect in *oskar* mRNA localisation, we performed *in situ* hybridisations on *wel* mutant clones in the absence of GFP-Staufen, and observed a very similar range of phenotypes (Fig. 1E-G). By contrast, the localisation of *bicoid* and *gurken* mRNAs was largely normal in *wel* mutant oocytes, suggesting that the defects are focussed on the posterior of the oocyte (Fig. 2).

We used deficiencies to map *wellman* to a 180 kb region in 93C1-D4 based on the lethality of both *wel* alleles in trans to *Df(3R)e-R1*, but not *Df(3R)Exel6272* and *Df(3R)ED6058* (Parks et al., 2004; Ryder et al., 2007). Crossing *wel* alleles to available mutants in this region revealed that they failed to complement the lethality of a null allele of *slmb*, *slmb*^00295^, leading us to rename the two *wel* alleles as *slmb*^8A6-5^ and *slmb*^9H4-17^ (Jiang and Struhl, 1998). Slmb is an F-box protein that functions as the substrate recognition subunit of the SCF (SKP1/Cullin/F-box protein) ubiquitin ligase, which targets proteins for degradation by the proteasome (Cardozo and Pagano, 2004). Slmb interacts with the SCF complex via its N-terminal F-box motif and binds to its substrates through seven WD40 repeats. Sequencing revealed that *slmb*^9H4-17^ contains a nonsense mutation at amino acid 167, and it is therefore presumably a null allele that encodes a truncated protein lacking the substrate interaction domains (Fig. 1I). The other allele, *slmb*^8A6-5^, has an alanine-to-valine substitution at amino acid 492 (Fig. 1I). Both alleles cause lethality during the first larval instar as homozygotes and produce a similar spectrum of phenotypes in germline clones, suggesting that *slmb*^8A6-5^ also behaves as a null. This might be because this allele introduces a bulkier side chain into a conserved amino acid adjacent to the key tyrosine residue that interacts with the substrates (Wu et al., 2003).

Germline clones of *slmb* null alleles have previously been found to cause a low frequency (17%) of morphological defects, but their effects on axis formation were not examined (Muzzopappa and Wappner, 2005). To confirm that the *oskar* mRNA localisation phenotype of *slmb*^8A6-5^ and *slmb*^9H4-17^ clones was due to the loss of Slmb, we took advantage of the fact that *slmb* transheterozygous adults can be generated by expressing Slmb under the control of a heat shock promoter (hs-Slmb) from the first instar larva until pupariation (Grima et al., 2002). Using this strategy, we generated *slmb*^8^/*slmb*^9H4-17^ transheterozygous ovaries, and observed a similar spectrum of Staufen localisation defects to those of *slmb*^9H4-17^ germline clones, although these were somewhat less penetrant, presumably due to the perdurance of Slmb expressed from the heat shock promoter (Fig. 1D,H). These phenotypes were largely rescued by expressing GFP-Slmb in the germline, confirming that mislocalisation of *oskar* mRNA and Staufen is caused by the specific loss of Slmb activity in the germline. A proportion of *slmb* mutant egg chambers escape the polarity defect and develop a later phenotype that we have already described elsewhere, in which short Oskar protein overaccumulates, leading to increased pole cell number and embryonic patterning defects (Morais-de-Sa et al., 2013).

### Slmb is required for the posterior recruitment of Par-1

**Fig. 3.**
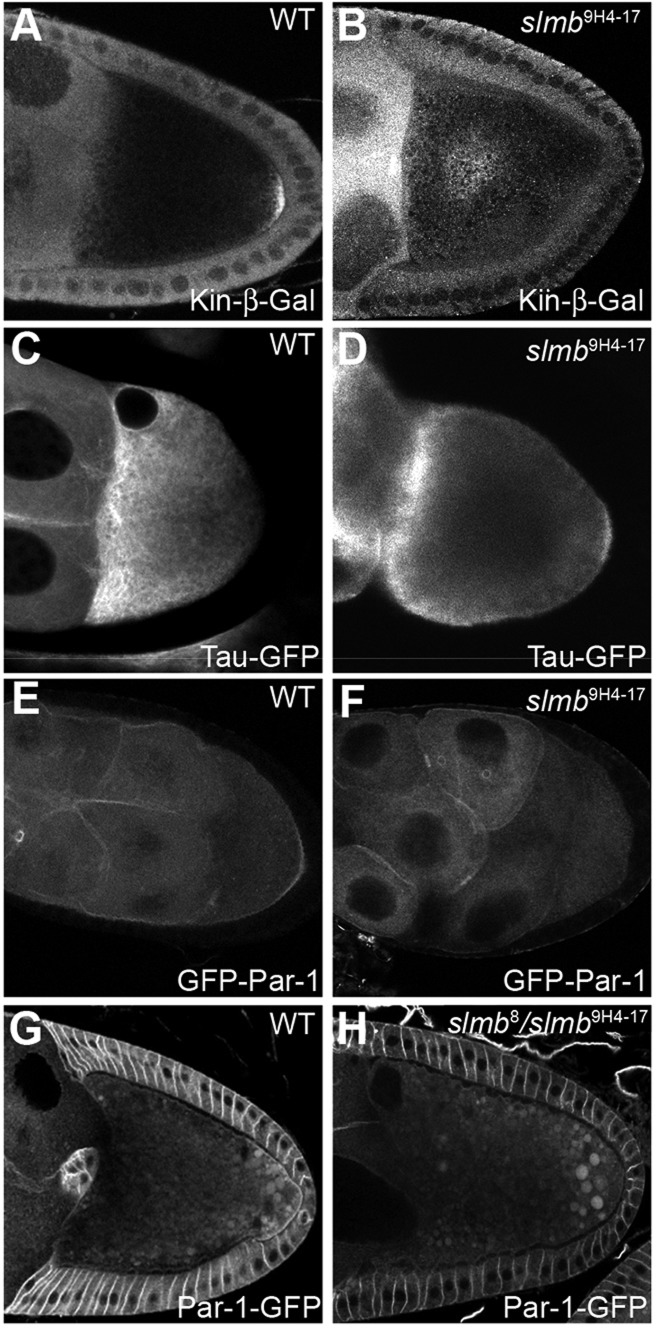
**Slmb is required for oocyte polarisation.** (A,B) Kinesin-β-galactosidase (Kin-β-gal) visualised with an antibody against β-gal. Kin-β-gal accumulates at the posterior in wild-type (A) but is diffusely localised to the centre of *slmb*^9H4-17^ germline clone oocytes (B). (C,D) Microtubule organisation in wild-type (C) and *slmb*^9H4-17^ (D) stage 9 egg chambers visualised with Tau-GFP. (E,F) Egg chambers expressing matα4:GFP-Par-1N1S. GFP-Par-1 forms a posterior crescent in wild-type oocytes (E) but fails to localise to the posterior of most *slmb*^9H4-17^ germline clone oocytes (F). (G,H) Par-1-GFP (expressed from a protein trap insertion in the endogenous *par-1* locus) forms a posterior crescent in wild-type oocytes (G) but is usually absent from the posterior of hs-Slmb, *slmb*^8^/*slmb*^9H4-17^ oocytes (H).

The *oskar* mRNA localisation phenotype of *slmb* germline clones resembles that of *par-1* mutants, which fail to repolarise the oocyte microtubule cytoskeleton (Shulman et al., 2000). We examined the organisation of the microtubules by expressing a constitutively active form of the plus end-directed microtubule motor protein kinesin, fused to β-galactosidase (Kin-β-gal) (Clark et al., 1994). In wild type, Kin-β-gal localises to the posterior pole of the oocyte at stage 9 by moving along microtubules to the region with the highest ratio of plus ends to minus ends (Fig. 3A). By contrast, Kin-β-gal accumulates in the centre of most *slmb* mutant oocytes, indicating that the plus ends are enriched in this region (Fig. 3B). Microtubule organisation can also be visualised by directly labelling them with GFP fused to the microtubule-binding protein Tau (Micklem et al., 1997). Tau-GFP labels an anterior-to-posterior gradient of microtubules in wild-type oocytes, with the highest density at the oocyte anterior where most microtubules are nucleated or anchored, and with little or no signal at the posterior (Fig. 3C). Some *slmb* mutant oocytes, by contrast, show strong Tau-GFP signal at the posterior pole of the oocyte, indicating that the *oskar* mRNA phenotype is caused by defects in the AP polarisation of the microtubule cytoskeleton (Fig. 3D).

The correct organisation of the microtubules depends on the recruitment of Par-1 to the posterior cortex of the oocyte in response to the polarising follicle cell signal (Doerflinger et al., 2006). We expressed a GFP-Par-1N1S transgene in *slmb* mutant germline clones to determine whether Slmb functions upstream or downstream of Par-1 in microtubule organisation. In wild type, GFP-Par-1 becomes enriched at the posterior cortex of the oocyte from stage 7 onwards and provides the earliest marker for the repolarisation of the oocyte (Fig. 3E). This posterior GFP-Par-1 crescent fails to form in *slmb*^9H4-17^ oocytes (Fig. 3F). There is also no posterior GFP-Par-1 crescent in 38% (*n*=26) of rescued *slmb*^8^/*slmb*^9H4-17^ adults, which correlates well with the penetrance of the Staufen localisation defect in this mutant combination. This result was confirmed by examining the localisation of a Par-1-GFP protein trap insertion that is expressed at endogenous levels, which also failed to form a posterior crescent in *slmb*^8^/*slmb*^9H4-17^ (Fig. 3G,H). Thus, Slmb is required in the germline for the posterior recruitment of Par-1, suggesting that it acts downstream of the signal from the posterior follicle cells to polarise the oocyte.

### Slmb limits the size of the apical domain in epithelial cells

**Fig. 4.**
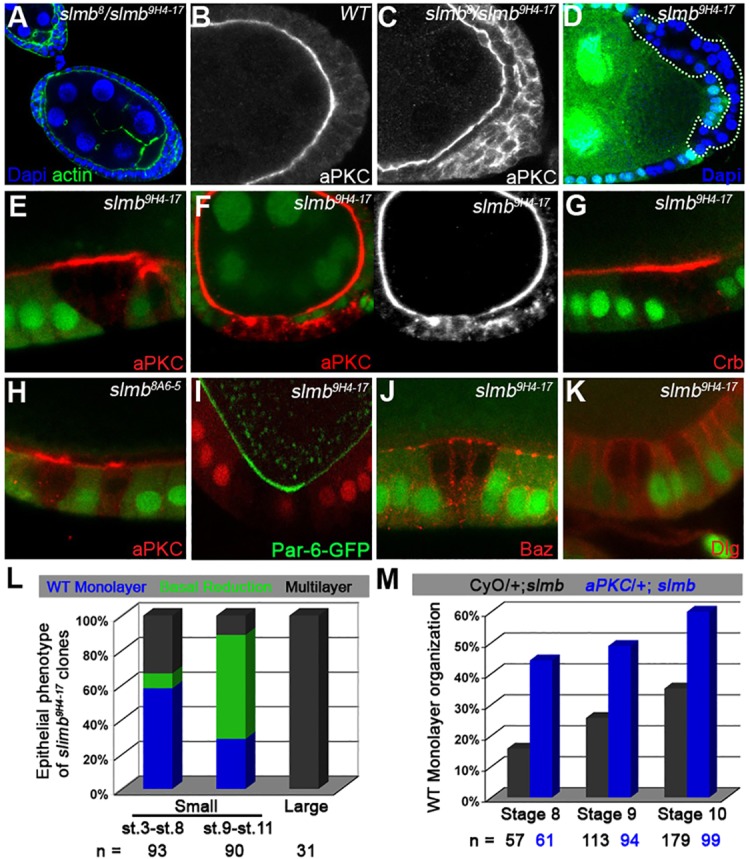
**Slmb regulates follicle cell organisation and polarity.** (A) The follicular epithelium is disrupted in hs-Slmb, *slmb*^8^/*slmb*^9H4-17^ egg chambers 5 days after the last period of Slmb expression. Multiple layers of epithelial cells form at the posterior and anterior of mutant egg chambers. DAPI (blue) marks the nuclei and actin (green) reveals overall morphology. (B) A wild-type egg chamber showing the localisation of aPKC at the apical side of the follicle cells, which form a uniform monolayer around the oocyte. (C) Magnification of the posterior end of an hs-Slmb, *slmb*^8^/*slmb*^9H4-17^ egg chamber 5 days after the last period of Slmb expression. aPKC remains apical in the cells that contact the germline, but is distributed in the cytoplasm and along the cortex of the unpolarised cells that no longer contact the germline. (D) A large *slmb*^9H4-17^ clone forming a double-layered epithelium (outlined) stained with DAPI. (E-K) Follicle cell clones of *slmb*^9H4-17^ (E-G,I-K) and *slmb*^8A6-5^ (H) marked by the absence of GFP (green) or RFP (red in I), stained in red for aPKC (E,F,H), Crb (G), Baz (J) and Dlg (K), and in green for Par-6-GFP expressed from a genomic rescue construct (I). Cells in small mutant clones have reduced basal domains with higher levels of aPKC, Par-6 and Crb apically, but show normal localisation of Baz and Dlg. (F) A large *slmb* clone with a disrupted epithelial organisation and aPKC around the cortex. (L) Quantification of the phenotypes of *slmb*^9H4-17^ follicle cell clones. Clones of fewer than 10 cells were scored as small. (M) Removing one copy of *aPKC* partially rescues the *slmb* mutant phenotype. Quantification of the percentage of stage 8, 9 and 10 egg chambers with an intact epithelial monolayer in CyO/+; hs-Slmb, *slmb*^8^/*slmb*^9H4-17^ and *aPKC^K06403^*/+; hs-Slmb, *slmb*^8^/*slmb*^9H4-17^, dissected 4 days after hs-Slmb induction.

*slmb*^8^/*slmb*^9H4-17^ mutants rescued by the expression of hs-Slmb during larval development also show defects in the organisation of epithelial follicle cells. These cells normally form a simple epithelial monolayer with their apical surfaces facing the germ cells. In *slmb*^8^/*slmb*^9H4-17^ egg chambers, the cells form multiple layers (Fig. 4A). This phenotype is reminiscent of that produced by mutants that affect epithelial polarity, and we therefore analysed the localisation of aPKC, a component of the apical polarity complex (St Johnston and Ahringer, 2010). aPKC localises normally to the apical surface of the mutant follicle cells that contact the germline, but shows a patchy distribution around the cortex of the cells that do not, which also lose their regular columnar arrangement (Fig. 4B,C). This suggests that Slmb is important for apicobasal polarity, but that an apical cue from the germline can compensate for reduced Slmb levels in most mutant cells (Tanentzapf et al., 2000).

Since the hs-Slmb-rescued *slmb* transheterozygotes show a weaker phenotype in the germline than *slmb* mutant clones, we also generated somatic clones of *slmb*^8A6-5^ and *slmb*^9H4-17^. The rare large clones that we recovered show a multilayered phenotype (Fig. 4D,L). Small clones also frequently show disruptions in epithelial polarity, even when the cells contact the germline (Fig. 4L). Clones comprising only a few mutant cells show a reduction of the basal domain and increased staining for the apical polarity proteins aPKC, Par-6 and Crumbs (Crb) (Fig. 4E,G-K). In larger clones, aPKC is also found throughout the cortex and cytoplasm, and the cells are often extruded from the epithelium (Fig. 4F). We also examined other polarity markers in *slmb* clones, including Baz, which marks adherens junctions, and Dlg (Dlg1 – FlyBase), which labels the lateral membrane (Morais-de-Sa et al., 2010; Woods et al., 1997). Both proteins show normal localisations in small clones with defective epithelial organisation, whereas aPKC and Par-6 levels are already elevated in small clones that still retain a normal shape (Fig. 4H-K). Thus, the increased levels of aPKC and Par-6 are not a consequence of disrupted epithelial organisation. This suggests that polarity defects in *slmb* mutants might result from overexpression of the Par-6/aPKC apical polarity complex, which disrupts the balance between the apical and basolateral domains. In support of this view, lowering aPKC levels by removing one copy of the gene partially rescues the multilayering phenotype of *slmb* transheterozygotes (Fig. 4M).

### The polarity defects of *slmb* mutants are not caused by overexpression of Arm or α-spectrin

Since Slmb ubiquitylates proteins to target them for degradation, loss of Slmb should lead to the overexpression of its substrates. This suggests that the epithelial phenotype results from the overexpression of one or more Slmb targets that regulate polarity. Although many Slmb substrates have been identified, these are mainly involved in signal transduction or cell cycle control (Fuchs et al., 2004). One possible candidate is Armadillo (Arm), which links E-cadherin to the actin cytoskeleton at adherens junctions as well as functioning in the Wingless pathway (Bienz, 2005). As expected, Arm is strongly overexpressed in *slmb* mutant follicle cells and germline clones (supplementary material Fig. S1A-D). This does not appear to be the cause of the polarity phenotype, however, as the overexpression of a stabilised form of Arm has no effect on epithelial organisation (supplementary material Fig. S1E-G). Furthermore, the strength of the germline polarity phenotype of *slmb* null mutant clones is not altered by removing one copy of *arm* (supplementary material Fig. S1I).

**Fig. 5.**
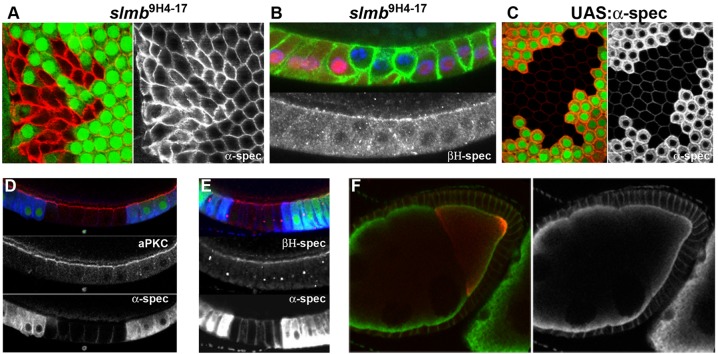
**α-spectrin overaccumulates in *slmb* mutant cells.** (A) Surface view of a large *slmb*^9H4-17^ clone marked by the absence of GFP (green), showing the strong increase in α-spectrin levels (red and as separate channel). (B) An *slmb*^9H4-17^ clone marked by the absence of nuclear RFP (red) stained for α*-*spectrin (green) and βH-spectrin (white, bottom panel). βH-spectrin levels are unchanged in the mutant clone. (C-E) Overexpression of α*-*spectrin in FLPout clones marked with GFP. Overexpressed α*-*spectrin (red) accumulates along the cell cortex and also in the cytoplasm (C). Overexpression of α*-*spectrin does not disrupt epithelial organisation, aPKC (red) localisation and stability (D) or βH-spectrin localisation and stability (E). (F) A wild-type egg chamber overexpressing α*-*spectrin in the germline driven by matα4-GAL4:VP16. α-spectrin (green) overexpression does not affect the posterior localisation of Stau (red).

We noticed that the levels of α-spectrin are also elevated in *slmb* mutant clones. α_2_/β_2_ spectrin tetramers are a major component of the cortical cytoskeleton that cross-links the plasma membrane to actin and tethers proteins in specific membrane domains (Knust, 2000). The *Drosophila* spectrin cytoskeleton is polarised with α_2_/β_2_ tetramers marking the basolateral membrane and α_2_/βH_2_ spectrin localising apically, where βH-spectrin is recruited by Crb (Lee et al., 1997; Medina et al., 2002; Thomas and Kiehart, 1994). *slmb* mutant clones show a dramatic increase in the levels of α-spectrin, whereas βH-spectrin is not significantly increased (Fig. 5A,B). This suggests that α-spectrin is normally ubiquitylated by the SCF complex and targeted for degradation. Since α-spectrin mutants disrupt the organisation of the follicular epithelium, we tested whether its overexpression could explain the polarity defects of *slmb* mutants. However, the expression of very high levels of α-spectrin in the follicle cells using the UAS-Gal4 system had no effect on epithelial polarity or the levels and apical localisation of aPKC or βH-spectrin (Fig. 5C-E). In addition, α-spectrin overexpression in the germline did not disrupt oocyte polarity (Fig. 5F). Thus, the upregulation of α-spectrin appears to be unrelated to the increased localisation of apical polarity factors and the expansion of the apical domain in *slmb* mutant clones.

### Slmb regulates the levels of Par-6 and aPKC

**Fig. 6.**
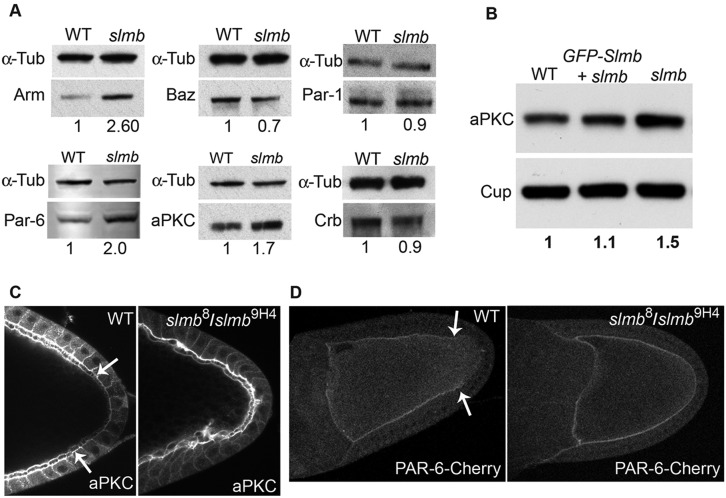
**Slmb regulates the distribution and levels of aPKC and Par-6.** (A) Western blots of ovary protein extracts from *w^−^* (WT) and hs-Slmb, *slmb*^8^/*slmb*^9H4-17^ (*slmb*) females 5 days after the last hs-Slmb induction probed for Arm, Baz, Par-6, aPKC, Par-1 and Crb. The levels of Arm increase in the *slmb* mutant extract compared with the α-Tubulin loading control, as do those of Par-6 and aPKC, whereas Baz, Par-1 and Crb levels are slightly reduced. For Par-1, the blot was probed with anti-GFP using extracts from ovaries from a Par-1-GFP protein trap line, in which a *GFP* exon is inserted in the middle of the endogenous *par-1* locus. (B) Western blot of ovary protein extracts from *w^−^* (WT), hs-Slmb, *slmb*^8^/*slmb*^9H4-17^ (*slmb*) and *nanos-Gal4VP16*; UASGFP-Slmb; hs-Slmb, *slmb*^8^/*slmb*^9H4-17^ (*GFP-Slmb*+*slmb*) probed for aPKC. Expression of GFP-Slmb in the germline reduces aPKC to almost wild-type levels. The same membrane was probed for Cup as a loading control. (C) Antibody staining of aPKC in a wild-type egg chamber and an hs-Slmb, *slmb*^8^/*slmb*^9H4-17^ egg chamber 4 days after the last heat shock. aPKC localises to the apical side of the follicle cells and around the lateral cortex of the oocyte in wild type, but is excluded from the posterior cortex (the region demarcated by white arrows). In the *slmb* mutant, aPKC extends around the posterior cortex of the oocyte. (D) Par-6-Cherry driven with *nanos-Gal4VP16* localises around the cortex of the oocyte in wild type, but is excluded from the posterior pole (arrows mark the end of the Par-6 domain). Par-6-Cherry is not excluded from the posterior cortex in *slmb*^8^*/slmb*^9H4-17^.

Since none of the obvious Slmb substrates could account for its polarity phenotype, we next considered the possibility that it targets the Par-6/aPKC complex for degradation. Western blots of adult hs-Slmb, *slmb*^8^/*slmb*^9H4-17^ egg chambers showed that the levels of aPKC and Par-6 increase significantly compared with wild type, whereas the levels of Baz and Crb are slightly reduced (Fig. 6A). Quantification indicates that Par-6 levels approximately double and aPKC levels are ∼60% higher than normal in *slmb* mutant egg chambers (Fig. 6A,B). This increase is almost completely suppressed by expressing GFP-Slmb in the germline. Thus, Slmb is required to limit the levels of the Par-6/aPKC complex, suggesting that it acts directly or indirectly to stimulate Par-6 or aPKC turnover. Since the levels of Baz and Crb are reduced in *slmb* mutants, it does not appear that the Baz/Par-6/aPKC complex as a whole is being regulated or that increased levels of Crb are recruiting more Par-6/aPKC to the cortex. Slmb must therefore target the Par-6/aPKC complex by a different mechanism.

Par-1 has also been reported to be a target of Slmb in neurons (Lee et al., 2012), and we therefore examined whether Par-1 levels are altered in hs-Slmb, *slmb*^8^/*slmb*^9H4-17^ egg chambers. Because the available antibodies against Par-1 do not work well on western blots, we examined whether loss of Slmb affected the amount of Par-1-GFP expressed from a protein trap line in which a *GFP* exon is inserted into the middle of the endogenous *par-1* locus (Lighthouse et al., 2008). Although one would expect the levels of a target of Slmb-dependent degradation to be elevated in an *slmb* mutant, Par-1-GFP levels are slightly reduced, indicating that it is not a substrate of the SCF^S^^lmb^ complex in the ovary (Fig. 6A).

### Slmb is required to exclude aPKC and Par-6 from the oocyte posterior cortex

In wild type, aPKC localises around the lateral cortex of the oocyte, but is excluded from the posterior cortex (Fig. 6C). By contrast, *slmb* mutant oocytes show a uniform cortical localisation of aPKC that spans the posterior pole (Fig. 6C). A Par-6-Cherry transgene can also be used as a marker for the Par-6/aPKC complex, as Par-6 binds to aPKC directly and colocalises with it in all other tissues (St Johnston and Ahringer, 2010). When expressed specifically in the germline, Par-6-Cherry is excluded from the posterior cortex of the oocyte at stages 8-9 in wild type, coincident with the posterior recruitment of Par-1 (Doerflinger et al., 2010). By contrast, Par-6-Cherry shows an almost uniform localisation around the cortex in *slmb* mutant germline clones (Fig. 6D). Thus, Slmb is required to remove Par-6 and aPKC from the oocyte posterior. This function of Slmb in controlling the asymmetric distribution of PAR-6 and aPKC in the oocyte differs from its role in the follicle cells, where it appears to simply restrict the levels of two polarity proteins to limit the size of the apical domain. Consistent with this, reducing the levels of aPKC by removing one copy of the gene does not significantly rescue the oocyte polarity phenotype of an *slmb* mutant (supplementary material Fig. S2).

### Overexpression of Lgl partially rescues the *slmb* polarity phenotype

**Fig. 7.**
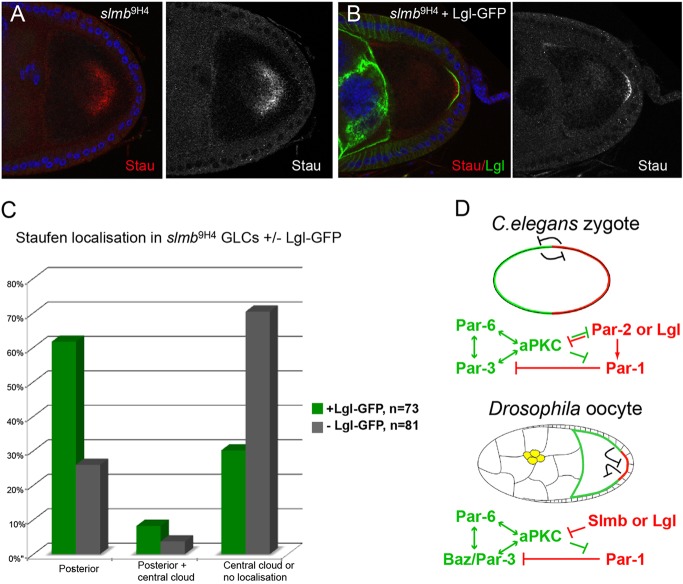
**Expression of Lgl-GFP in the germline rescues the *slmb* polarity phenotype.** (A) A *slmb*^9H4-17^ germline clone egg chamber showing the mislocalisation of Staufen (Stau, red) in a cloud in the centre of the oocyte. (B) A typical *slmb*^9H4-17^ germline clone egg chamber expressing Lgl-GFP. Lgl-GFP (green) localises normally to the posterior cortex of the oocyte and rescues the posterior localisation of Stau. (C) The frequency of Stau localisation phenotypes in *slmb*^9H4-17^ germline clones with and without expression of Lgl-GFP. (D) Model presenting Slmb as the functional counterpart of *C. elegans* PAR-2. Both proteins are required for the posterior exclusion of the aPKC complex and for the recruitment of Par-1, and both can be rescued by overexpressing Lgl. Border cells are shown in yellow.

Lgl might play a redundant role in the polarisation of the oocyte, since it localises to the posterior cortex and is a known antagonist of aPKC (Tian and Deng, 2008; Wirtz-Peitz et al., 2008). We examined the effects of overexpressing UAS-Lgl-GFP in *slmb* mutants. Expression of Lgl-GFP in the germline strongly suppresses the polarity phenotype of *slmb*^9H4-17^ germline clones, and the majority of oocytes show a posterior crescent of Lgl-GFP and normal posterior localisation of Staufen protein (Fig. 7A-C). Thus, Lgl appears to function independently of Slmb in the inactivation of the Par-6/aPKC complex at the posterior cortex of the oocyte, and can compensate for the loss of Slmb when overexpressed.

## DISCUSSION

Very little is known about how the posterior follicle cells signal to polarise the AP axis of the oocyte, except that signalling is disrupted when the germline is mutant for components of the exon junction complex, such as Mago nashi (Micklem et al., 1997; Newmark et al., 1997). Our results reveal that Slmb also plays an essential role in this pathway, where it acts to establish the complementary cortical domains of Baz/Par-6/aPKC and Par-1. Although Slmb might act in a variety of ways to establish this asymmetry, the observation that it regulates the levels of the Par-6/aPKC complex suggests a simple model in which Slmb directly or indirectly targets a component of the complex for degradation at the posterior of the oocyte. Since aPKC phosphorylates Par-1 to exclude the latter from the cortex, the degradation of aPKC would allow the posterior recruitment of Par-1, which would then maintain polarity by phosphorylating and antagonising Baz (Benton and St Johnston, 2003; Hurov et al., 2004; Suzuki et al., 2004). Indeed, this might explain the observation that Par-6 is excluded from the posterior cortex before Baz (Doerflinger et al., 2010). The polarisation of the oocyte therefore appears to occur in two phases. During the initiation phase, Slmb removes the Par-6/aPKC complex from the posterior cortex to allow the recruitment of Par-1. Par-1 then maintains and reinforces this asymmetry by phosphorylating Baz to exclude it from the posterior cortex, thereby removing the cortical anchor for the Par-6/aPKC complex.

Slmb is usually recruited to its targets by binding to phosphorylated residues that lie 9-14 amino acids downstream from the ubiquitylated lysine (Wu et al., 2003). Although both aPKC and Par-6 contain several sequences that could serve as atypical Slmb binding sites, neither contains a classic Slmb-dependent degron sequence. It is therefore unclear whether the SCF^Slmb^ complex directly ubiquitylates either protein to target it for degradation or whether it targets another, unknown component of the complex that is required for the stability of Par-6 and aPKC. Nevertheless, this model leads to the prediction that the polarising signal from the follicle cells will induce the activation of a kinase that phosphorylates a Slmb substrate at the posterior of the oocyte, thereby triggering the local degradation of the Par-6/aPKC complex.

The demonstration that Slmb is required for the exclusion of the Par-6/aPKC complex from the posterior of the *Drosophila* oocyte raises interesting parallels with AP axis formation in *C. elegans*. Although *Drosophila* does not have an equivalent of the main symmetry-breaking step in the worm, in which a contraction of the actomyosin cortex removes the anterior PAR proteins from the posterior, the function of Slmb is analogous to that of PAR-2 in the alternative polarity induction pathway (Zonies et al., 2010). Both proteins act to remove the Par-6/aPKC complex from the posterior cortex to allow the posterior recruitment of Par-1, which then reinforces polarity by excluding Baz/PAR-3 by phosphorylation (Fig. 7D). Furthermore, the polarity phenotypes of both *slmb* and *par-2* mutants can be rescued by the overexpression of Lgl (Beatty et al., 2010; Hoege et al., 2010). Slmb and PAR-2 act by different mechanisms, since the former is a subunit of the SCF ubiquitin ligase complex and promotes the degradation of the Par-6/aPKC complex, whereas the latter functions by recruiting PAR-1 (Motegi et al., 2011). Nevertheless, it is intriguing that PAR-2 contains a RING finger domain that is typically found in ubiquitin ligases (Joazeiro and Weissman, 2000; Lorick et al., 1999), suggesting that it might have lost this activity during evolution.

## MATERIALS AND METHODS

### *Drosophila* strains and genetics

*wellman* was genetically mapped using a set of 3R deficiency stocks obtained from the Bloomington and Szeged *Drosophila* stock centres. Clonal analysis was performed with the FLP/FRT system using nuclear GFP as a marker of wild-type cells (Xu and Rubin, 1993) or using the dominant female sterile technique with the *FRT82 ovoD* chromosome (Chou and Perrimon, 1996). The transgenes and mutant alleles used were: *slmb*^8A6-5^ and *slmb*^9H4-17^ (this study); *hsSlmb*, *slmb*^8^ (Grima et al., 2002); *kin-β-GAL* (Clark et al., 1994); *Tau-GFP* (Micklem et al., 1997); *GFP-Stau* (Schuldt et al., 1998); a genomic *PAR-6-GFP* rescue construct (Wirtz-Peitz et al., 2008); *UASp:PAR-6-Cherry* (Doerflinger et al., 2010); Par-1-GFP trap line (Lighthouse et al., 2008); *mat-tub-GFP-PAR-1* and *UASp-GFP-PAR-1(N1S)* (Huynh et al., 2001); *UASt-ArmS10-Myc* (Pai et al., 1997); *UASp-α-spectrin* (this study) and *UASp-GFP-Slmb* (Morais-de-Sá et al., 2013). *matα4-GAL4:VP16* V32A and nanosGal4:VP16 were used to induce expression in the germline. To generate FLPout clones, UAS transgenes were crossed into *y,w, hsFlp; tub-FRT-cc-FRT-Gal4, UAS:GFP*. Lgl-GFP was expressed from the P{UASp-l(2)gl.GFP} construct (Tian and Deng, 2008). The construct was remobilised from its original location on the third chromosome onto the second chromosome to allow recombination with drivers located on the second chromosome. GeneSwitch inducible Gal4 drivers of two types were used: *Maternal alpha4 tubulin*-GeneSwitch drivers, which gave a moderate background level of Lgl-GFP, and weaker *vasa*-GeneSwitch drivers, which gave no expression unless induced with hormone (Osterwalder et al., 2001). All combinations of driver and Lgl-GFP transgene showed suppression of the Staufen mislocalisation phenotype of *slmb*^9H4-17^ when Lgl-GFP was present.

### Immunological techniques and *in situ* hybridisation

Immunofluorescence (IF) and western blots (WB) were performed using standard procedures with the following primary antibodies: rabbit anti-Stau [1/100 (St Johnston et al., 1991)], rabbit anti-PKCz [1/500 IF, 1/2000 WB (C-20, Santa Cruz Biotechnology)], mouse anti-Gurken [1/30; Developmental Studies Hybridoma Bank (DSHB)], mouse anti-Dlg (1/200; DSHB), rabbit anti-Par-6 [1/2000 WB (Petronczki and Knoblich, 2001)], rabbit anti-Baz [1/1000 IF, 1/5000 WB (Wodarz et al., 1999)], mouse anti-Crb (1/50; DSHB), mouse anti-Arm (1/100 IF, 1/2000 WB; DSHB), mouse anti-α-spectrin (1/100; DSHB), rabbit anti-βH-spectrin [1/500 (Thomas and Kiehart, 1994)] and mouse anti-α-tubulin (1/2000 WB; DM1A, Sigma). Actin was visualised with Rhodamine-conjugated phalloidin (Invitrogen). *In situ* hybridisations for *oskar* and *bicoid* mRNA were performed as previously described (Doerflinger et al., 2006; Schnorrer et al., 2002). Imaging was performed with a Zeiss LSM510 confocal microscope with a 40× oil immersion lens (Plan-NeoFluor; NA 1.3). Images were processed using ImageJ (NIH) and Adobe Photoshop.

For quantitative western blots, the ovaries of 15 females of each genotype were lysed in 225 μl ice-cold RIPA buffer (Sigma) with protease inhibitors (Roche) and dissociated using a disposable pestle. The resulting lysate was passed through a 27G needle (BD Biosciences) five or six times to shear the DNA and spun at 13,000 rpm (15,700 ***g***) at 4°C for 10 min. The middle layer of the supernatant was removed and respun before adding an equal volume of 2× Laemmli buffer and heating for 10 min at 100°C. For Par-6 western blotting, we used anti-rabbit IgG (Alexa 790) and anti-mouse IgG (Alexa 680) secondary antibodies (Life Technologies) overnight at 1:10,000 in 5% milk and quantified the signal using a Licor Odyssey western blot scanner. For the other proteins, we used anti-mouse or anti-rabbit IgG secondary antibodies conjugated to HRP (GE Healthcare) and the ECL or ECL Prime detection kit (GE Healthcare). The levels of aPKC, Par-6 and Baz on western blots were quantified by measuring the ratio of the signal relative to a control protein using Scion Image software or FIJI (ImageJ) and normalising to the wild-type ratio.

### Molecular biology

To sequence the *slmb* alleles, exons were individually amplified from genomic DNA and sequenced by Geneservice (Cambridge, UK). To clone *UASp-α-spectrin*, the *α-Spectrin* genomic region including all the coding sequence was amplified from *Drosophila* genomic DNA with the primer pair 5′-TATAGGTACCATGGAGAACTTTACACCC-3′ and 5′-TATAGC-GGCCGCTTAGTTCTGGAACAGCGTTC-3′, and cloned into the *Kpn*I/*Not*I sites of the pUASp-PL vector (Rørth, 1998). This transgene was introduced into flies by standard germline transformation.

## Supplementary Material

Supplementary Material
