# Supplementary material for: Slmb antagonises the aPKC/Par-6 complex to control oocyte and epithelial polarity
Source: Development. 2014 Aug;141(15):2984–92. doi: 10.1242/dev.109827 (PMC4197659; doi:10.1242/dev.109827)
Supplement: Supplementary Material [file supp_141_15_2984__index.html]

Slmb antagonises the aPKC/Par-6 complex to control oocyte and epithelial polarity — Supplementary Material 

# Slmb antagonises the aPKC/Par-6 complex to control oocyte and epithelial polarity

## DEV109827 Supplementary Material

**Files in this Data Supplement:**

- **Supplementary Material**
