# Supplementary material for: Slmb antagonises the aPKC/Par-6 complex to control oocyte and epithelial polarity
Source: Development. 2014 Aug;141(15):2984–92. doi: 10.1242/dev.109827 (PMC4197659; doi:10.1242/dev.109827)
Supplement: Supplementary Material [file supp_141.15.2984_DEV109827.pdf]

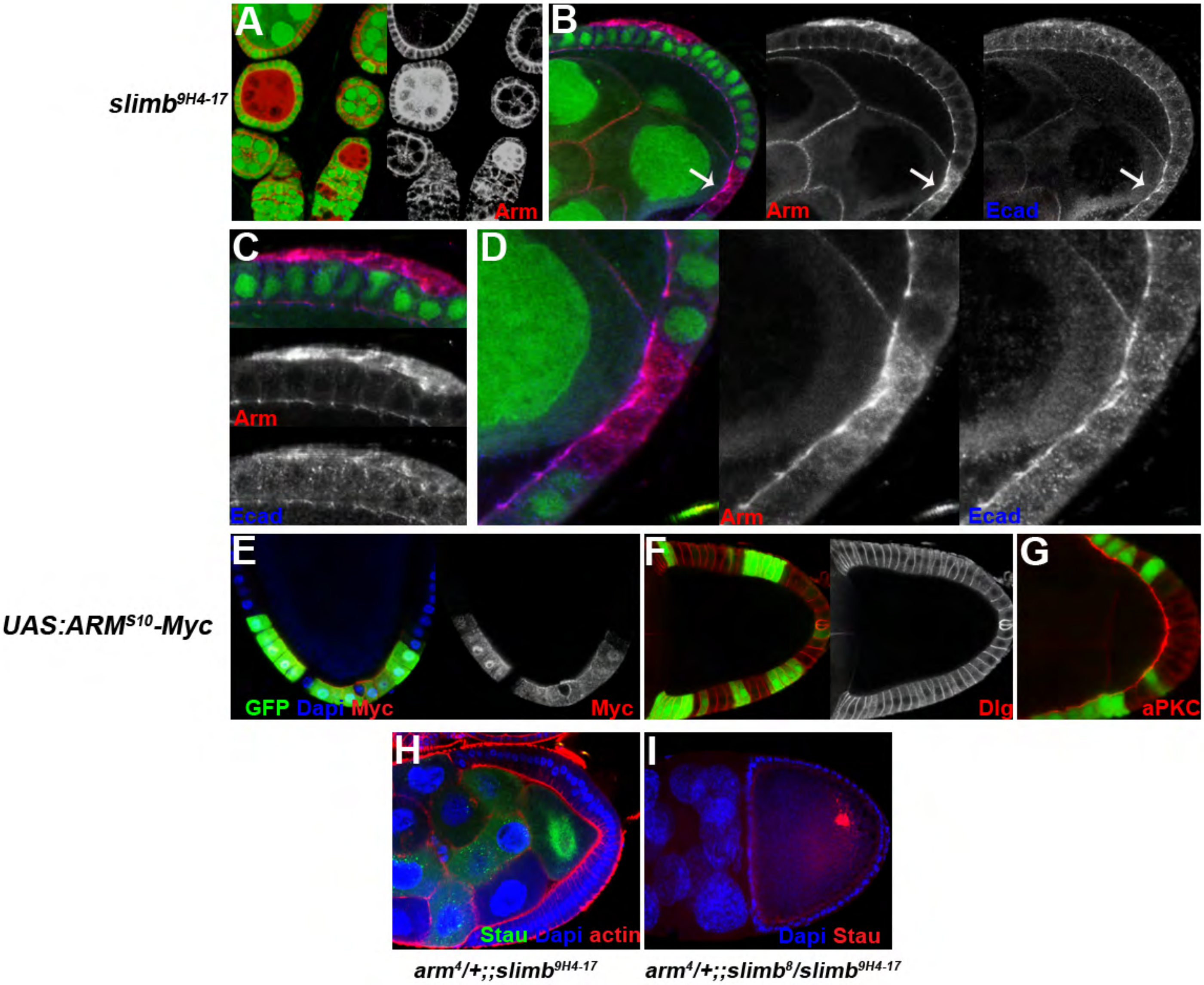

Figure S1: Slmb does not organize polarity via Armadillo regulation. (A-D) *slmb*$^{9H4-17}$ clones marked by the absence of GFP. Arm (red) is stabilized in both *slmb* mutant germ cells (A) and follicle cells (B-D). (C) A close up of the follicle cell clone at the top of (B) that has been extruded from the epithelial monolayer, whereas (D) shows a small clone that has remained in the epithelial monolayer (D). Arm is dramatically stabilized in both clones, but the adherens junctions, marked by DE-Cadherin (blue) are not enlarged, despite being misorganized in (C). (E-G) Overexpression of a stabilized form of Arm (Arm^{S10}-Myc) in FLPout clones marked with GFP. (E) Arm^{S10}-Myc is detected in GFP marked cells with an anti-c-Myc (red) antibody. (F,G) Overexpression of Arm^{S10} does not induce polarity defects as seen by the wildtype localization of Dlg (red in F) or aPKC (red in G). (H,I) Removing one copy of Armadillo in *arm*^{4} heterozygotes (an amorphic allele) does not rescue the Staufen localization defect of *slmb*$^{9H4-17}$ (H) or *slmb*^{8}/*slmb*$^{9H4-17}$ (I; 83% defective localization, n=122).

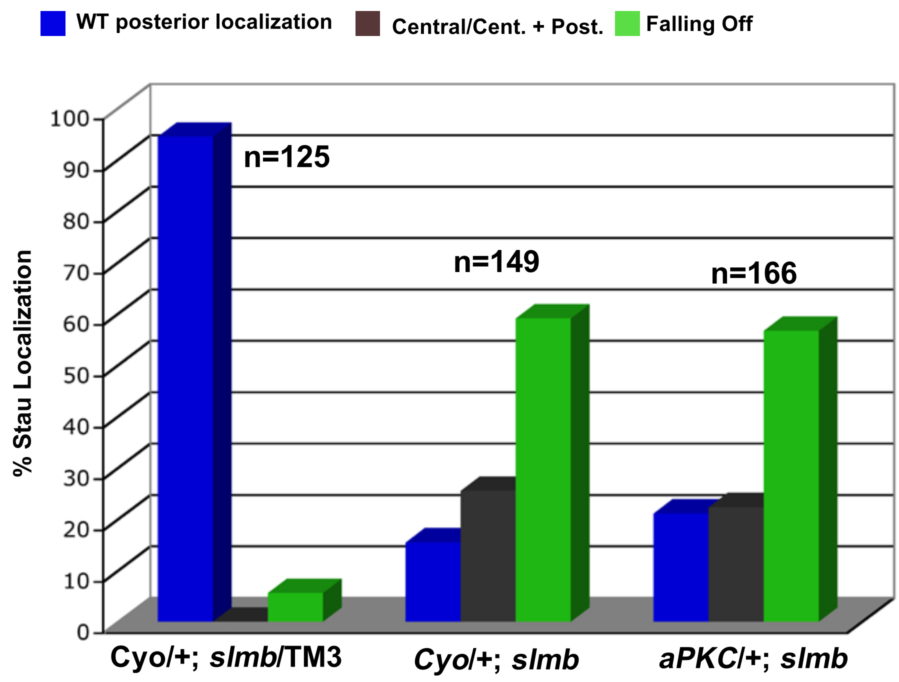

Figure S2: Reducing aPKC levels does not rescue the oocyte polarity phenotype of *slmb* mutants. The percentage of stage 9 and 10 oocytes with normal localisation of Staufen to the posterior pole (blue), with mislocalisation to the centre of the oocyte with or without some posterior localisation (grey), and with Staufen appearing to fall off the posterior pole (green) in CyO/+; hs-Slmb, *slmb*^{8}/+ (heterozygous control); CyO/+; hs-Slmb, *slmb*^{8}/*slmb*$^{9H4\text{-}17}$ and *aPKC*^{K06403}/+; hs-Slmb, *slmb*^{8}/*slmb*$^{9H4\text{-}17}$, dissected 4 days after hs-Slmb induction. Heterozygosity for *aPKC* does not significantly change the penetrance of the polarity defects in *slmb* mutants.
